# Supplementary material for: Diameter-independent skyrmion Hall angle observed in chiral magnetic multilayers
Source: Nat Commun. 2020 Jan 22;11:428. doi: 10.1038/s41467-019-14232-9 (PMC6976618; doi:10.1038/s41467-019-14232-9)
Supplement: Supplementary file 2 — Description of Additional Supplementary Files [file 41467_2019_14232_MOESM2_ESM.pdf]

Description of Additional Supplementary Files

File name: Supplementary Movie 1

Description: Raw scanning transmission x ray microscopy images taken at 0 mT. Two current pulses were applied between each frame.

File name: Supplementary Movie 2

Description: Raw scanning transmission x ray microscopy images taken at 0 mT second measurement series. Two current pulses were applied between each frame.

File name: Supplementary Movie 3

Description: Raw scanning transmission x ray microscopy images taken at -0.5 mT. Two current pulses were applied between each frame.

File name: Supplementary Movie 4

Description: Raw scanning transmission x ray microscopy images taken at -1.0 mT. Two current pulses were applied between each frame.

File name: Supplementary Movie 5

Description: Raw scanning transmission x ray microscopy images taken at -1.5 mT. Two current pulses were applied between each frame.

File name: Supplementary Movie 6

Description: Raw scanning transmission x ray microscopy images taken at -2.0 mT. Two current pulses were applied between each frame.

File name: Supplementary Movie 7

Description: Raw scanning transmission x ray microscopy images taken at -2.5 mT. Two current pulses were applied between each frame.

File name: Supplementary Movie 8

Description: Raw scanning transmission x ray microscopy images taken at -3.0 mT. Two current pulses were applied between each frame.

File name: Supplementary Movie 9

Description: Raw scanning transmission x ray microscopy images taken at -3.5 mT. Two current pulses were applied between each frame.

File name: Supplementary Movie 10

Description: Raw scanning transmission x ray microscopy images taken at -4.0 mT. Two current pulses were applied between each frame.
